# An urban-level prediction of lockdown measures impact on the prevalence of the COVID-19 pandemic

**DOI:** 10.1186/s41118-022-00174-6

**Published:** 2022-09-05

**Authors:** Saeid Pourroostaei Ardakani, Tianqi Xia, Ali Cheshmehzangi, Zhiang Zhang

**Affiliations:** 1 Department of Computer Science, University of Nottingham, Ningbo, 315100 China; 2 Department of Architecture and Built Environment, University of Nottingham, Ningbo, 315100 China

**Keywords:** Lockdown, Machine learning, COVID-19, Predictive analysis

## Abstract

The world still suffers from the COVID-19 pandemic, which was identified in late 2019. The number of COVID-19 confirmed cases are increasing every day, and many governments are taking various measures and policies, such as city lockdown. It seriously treats people’s lives and health conditions, and it is highly required to immediately take appropriate actions to minimise the virus spread and manage the COVID-19 outbreak. This paper aims to study the impact of the lockdown schedule on pandemic prevention and control in Ningbo, China. For this, machine learning techniques such as the K-nearest neighbours and Random Forest are used to predict the number of COVID-19 confirmed cases according to five scenarios, including no lockdown and 2 weeks, 1, 3, and 6 months postponed lockdown. According to the results, the random forest machine learning technique outperforms the K-nearest neighbours model in terms of mean squared error and R-square. The results support that taking an early lockdown measure minimises the number of COVID-19 confirmed cases in a city and addresses that late actions lead to a sharp COVID-19 outbreak.

## Introduction

As of December 2019, the world is experiencing a major pandemic, known as the novel coronavirus disease 2019 (COVID-19). The disease was rapidly spread across the globe, which is still ongoing at the time this paper is written (June 2022), with an uncertain end. According to the World Health Organization (WHO) COVID-19 (2021), there have been 223, 022, 538 confirmed COVID-19 cases and 4, 602, 882 deaths globally reported until September 2021. As Sen-Crowe et al. (2020) reports, COVID-19 is currently the third leading cause of daily death in the world.

Human-to-human close contact plays a key role in the pandemic outbreak. The virus is transmitted to a person if they live close to infected ones. Therefore, governments usually introduce particular social policies and/or take proper actions, such as travel and work restrictions, quarantines, curfews, and vaccinations to protect society and prevent the virus from spreading. For example, citizens have been recommended by several actions such as wearing masks and social distancing to minimise virus spread and control the outbreak (Surveillances, 2020). However, taking late or unplanned actions worsen the outbreak situation, especially in crowded and/or rural regions (Biau et al. 2012).

Lockdown is an efficient and promising measure to manage and prevent COVID-19 outbreak, especially if the lockdown schedule is properly analysed and planned (Kuo & Fu, 2021). The best-fitted lockdown schedule can be calculated and/or predicted according to people’s mobility pattern/trend (Kuo & Fu, 2021). However, there is still no common agreement on the start time and length of the lockdown period to effectively restrict the virus spread with minimised impact on the people’s social activities and events. If this is not accurately scheduled and implemented, the COVID-19 epidemic might be rebounded even after long-term lockdowns. For example, France took a national lockdown action on March 17, 2020, that resulted in a reduced number of confirmed cases by 77% from March to May. However, the number of COVID-19 cases sharply increased, and the country coped with a critical situation again after the lockdown measure was lifted in May 2020 (Kiem et al., 2021).

This paper focuses on the impact of lockdown measures on pandemic prevention and control in Ningbo city, Zhejiang province, China^1^. Ningbo lockdown was different with other countries as the rigorous policy measures for ‘China-style lockdown’ limited the city’s operations significantly. It led to the closure of intra-city and intra-provincial road transportation networks and railway transportation systems. Such measures also limited the number of times people could go outside their compounds and houses and closed all primary operations, secondary industries, and public activities. However, the time factor plays a significant part in the effectiveness of such lockdown measures. Hence, this analytical study provides five lockdown scenarios, each of which lasts four weeks, that could highlight the impact of road mobility on the COVID-19 outbreak and the number of confirmed cases in Ningbo.

Road mobility includes any sort of transportation that requires road networks in and out of the city, including highways, motorways, and secondary road networks between cities. The overarching network affects private car mobility, goods mobility, logistics for services and supplies, and public transportation with coaches and buses. During the COVID-19 pandemic in China, the government suspended all inter-provincial road, air and train transportation. Hence, the incoming flow of people into Ningbo city includes the flow of people travelling by car and on public transportation. The flow of people includes workers and labours, Ningbo residents, and people from regional businesses, trades, and industries.

The Pearson technique (Zhi et al., 2017) is used to measure the correlation between COVID-19 confirmed cases and road mobility. This approach forms a correlation matrix to highlight the impact of road transportation (i.e., from other Zhejiang cities to Ningbo) on the number of COVID-19 confirmed cases in Ningbo. Random Forest (Iannace et al., 2019) and K-Nearest Neighbors (Imandoust & Bolandraftar, 2013) machine learning techniques are used to explore data patterns and predict COVID-19 outbreak behaviour (i.e., number of confirmed cases according to road mobility ratio) based on lockdown’s start date. The time factor is taken into the account as an important variable for the implementation of lockdown measures. The machine learning models are trained using the COVID-19 report, and road mobility datasets, named (AutoNavi Map, 2021) and DXY.DX Doctor (Covid-19 confirmed cases, 2021), to predict the behaviour of COVID-19 outbreak based on five lockdown scenarios. A cross-validation approach is used to evaluate and compare the prediction methods.

The study’s novelty is based on an evaluative approach to multiple scenarios, using the time factor against lockdown measures implementation. It models multiple scenarios against an original lockdown scenario at the beginning of the ongoing COVID-19 pandemic. This approach helps to verify time against the effectiveness of lockdown measures, which differs from existing mobility-based modelling studies. This study differs from the methodological approach and the case study selection, intending to study the correlation between mobility and higher infection rates. The study contributes to existing debates on the nexus between mobility, outbreak spread, and time-variable for lockdown measures implementation. Unlike existing research studies, this study uses one city case with scenarios against itself, which helps to have a more accurate understanding of how the outbreak spread could be controlled by having earlier travel restrictions as part of the outbreak containment process. Moreover, the model uses multiple methods to increase the accuracy of results and understand the comparative scenarios. The key contributions of this research are outlined below:

1. To design a data-driven analysis aiming to highlight the correlation between the number of COVID-19 confirmed cases and mobility ratio according to a case study scenario.
2. To train two machine learning models to predict the impact of the lockdown measure on COVID-19 pandemic prevention and control based on five scheduling scenarios.
3. To evaluate and compare machine learning-enabled prediction analysis to figure out the best-fitted prediction model.
4. To analyse and propose an accurate lockdown scheduling plan to minimise the COVID-19 outbreak.

The rest of the paper is organized as the following: Related works section reviews the literature and outlines state-of-the-art data analysis approaches in this field of research. Data and methodology section focuses on the research methodology by introducing data analysis and machine learning techniques and approaches. Results and discussion section discusses the results and outlines the key research findings. It reports the machine learning prediction results to highlight the correlation between road mobility ratio and COVID-19 confirmed cases in Ningbo according to five lockdown scenarios. Finally, Conclusion and future work section summarizes the key findings of this research and addresses future works.

## Related works

Several research studies have been conducted to analyse and model the COVID-19 outbreak using data analysis and machine learning techniques in various contexts, such as the US, Europe, Australia, and Asia. This section aims to highlight their similarities, differences, and superiorities.

Li et al. (2021) discuss the correlations between people’s mobility, COVID-19 confirmed or death cases, and outbreak presentation and control policies, including public event cancellation, travel control, and public transportation restriction in the US. The impact of the policies on mobility and the number of COVID-19 cases is investigated using a regression technique. The results show that the social policies minimise public activities and control large-scale gatherings, resulting in significantly reduced mobility and the number of COVID-19 cases.

Kuo and Fu (2021) use machine learning techniques, including Elastic Net (EN), Principal Components Regression (PCR), Partial Least Squares Regression (PLSR), K-Nearest Neighbours regression (KNN), Regression Tree (RT), Random Forest (RF), Gradient Boosted Tree (GBM), and 2-layer Artificial Neural Network (ANN) models to predict the impact of lockdown measure on the virus outbreak. Accordingly, county-level populations, environment parameters, and mobile data are collected and analysed. Such an analytical approach addresses lockdown measures’ capacity to minimise the virus outbreak in New York City, Cook County, and Illinois.

Iacu et al. (2020) propose a COVID-19 outbreak model in France through internal and external mobile network data analysis of the Haut-Rhin mobile communication centre. A quadratic regression model is proposed to monitor the human mobility routes and model the dynamics of the early spread of the virus. The results show a strong correlation between the number of COVID-19 death cases and Haut-Rhin mobile communications before a national lockdown. They also conducted research with the same methodology in Italy and achieved similar results (Iacus et al., 2020). Moreover, they use a regression model to study the correlation between IgG SARS- Cov-2 antibody and population mobility data in Spain (Iacus et al., 2020). The results show that the number of people with positive IgG is correlated with the mobility ratio. It supports a low population immunity against the virus and addresses that the pandemic outbreak continues if mobility is uncontrolled and lockdown measures are not properly taken.

Hakim et al. (2021) propose a research program to study and compare the behaviour of the COVID-19 spread in Hong Kong, Singapore, Japan, and Australia. They obtain seven COVID-19 control and prevention policy measures, including gathering restrictions, international travel limitations, passenger screening, traveller isolation/quarantine, school closure, and business closure which the governments report on public media and government official website. This research reports that community mobility decreases in Australia, Japan, and Singapore, while remaining unchanged in Hong Kong. It collects the number of new daily COVID-19 confirmed cases to monitor and analyse the outbreak situation in each region. According to the result, the number of COVID-19 confirmed cases in Singapore increased despite having home quarantine.

Fraser and Aldrich (2020) utilise daily COVID-19 confirmed cases data and user’s mobility on Facebook to study the behaviour of pandemic/outbreak in Japan. The results support that communities with close social connections are more susceptible to COVID-19, whereas the rate of confirmed cases is sharply reduced in public due to national-level mobility restrictions.

Prakash et al. (2020) analyse COVID-19 data in India using machine learning techniques. They utilise a correlation matrix to recognise the dataset features that impact the COVID-19 spread. The results indicate that people in the age groups of $20-30$, $30- 40$, and $40-50$ are usually at the COVID-19 risk compared to others. According to this research, Random Forest is the best-fitted machine learning model to conduct a prediction analysis.

This literature review uncovers the impact of lockdown measures on the COVID-19 outbreak. However, it still lacks a data-driven analysis to study and predict the effect of the mobility restrictions on pandemic prevention according to different lockdown scenarios in terms of the start time. This research aims to resolve the existing drawback by proposing a predictive plan to schedule the social restrictions and analysing the impact of various lockdown scheduling scenarios on the virus outbreak.

## Data and methodology

**Fig. 1:**
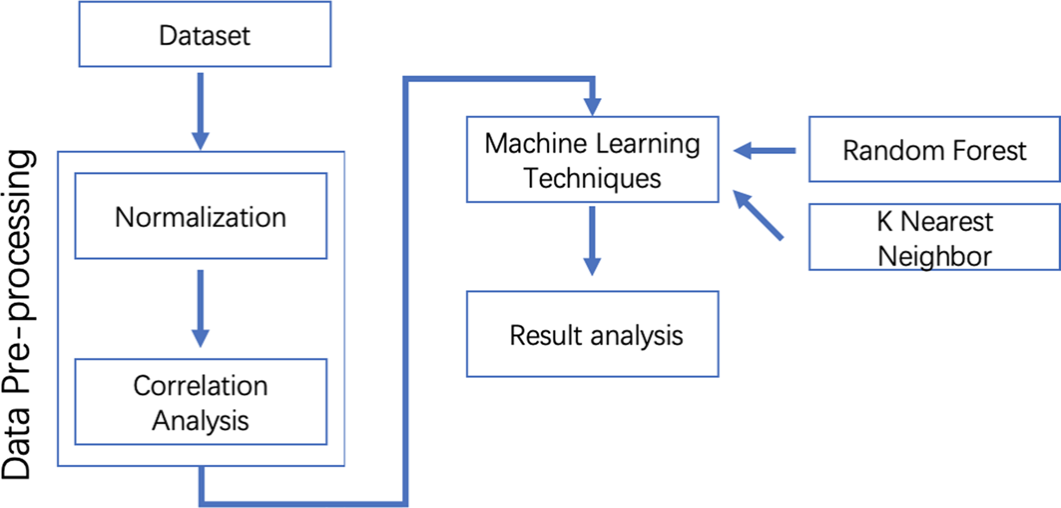
Research methodology diagram

This section introduces the case study and describes the research methodology by introducing the dataset verification, data pre-processing, feature selection, correlation method, and machine learning techniques. Figure 1 depicts a conceptual diagram for the research methodology. There are three key steps to form this data-driven research: data pre-processing (i.e., normalization and correlation analysis), machine learning model training for prediction analysis, and cross-validation.

### The case study

The study’s scope is related to the impact of lockdown measures and schedules on the COVID-19 outbreak. It focuses on the urban-level impacts of the lockdown measures in different scenarios, mainly to study the critical aspect of the ‘time of lockdown’ in the first few weeks of the COVID-19 pandemic. As we focus on the urban level, the study intentionally excludes outbound mobility that impacts the spread of the disease outside the city boundaries. As the study’s case study context is the scale of a city, we only need to consider the inbound mobility data that affects the city’s situation related to the prevalence of the pandemic or disease spread.

Ningbo city is a case study to understand the impact of lockdown schedules on pandemic prevention and control. Ningbo suffered a rapid increase in the number of confirmed cases, although the local government then immediately took several primitive actions (e.g., social distancing) Roosa et al. (2020) to manage the disease outbreak. Such early impact was because of a virus transmission chain that formed due to unmanaged road travels from other Zhejiang cities to Ningbo (Ying et al., 2020). Due to frequent transportation between Wuhan and Zhejiang, Zhejiang was initially the third most affected province (i.e., based on recorded infected cases) by the COVID-19 pandemic in China with 1, 205 confirmed cases by February 26, 2020 (Chong et al., 2019). However, the spread of COVID-19 in Zhejiang province was well controlled shortly after the outbreak due to immediate government policies and their implementation—i.e., mainly based on early lockdown measures. Zhejiang is the first province to announce the highest-level provincial public health emergency on January 23, 2020. It has suspended inter-provincial road transportation and cancelled all air and train travel from January 27, 2020. Moreover, Zhejiang cities took proper measures—mainly social distancing to control and prevent the pandemic. Therefore, Zhejiang successfully managed the COVID-19 outbreak by mid-to-late February.

Unlike many countries, China’s road transportation networks are monitored and managed at the municipal and provincial levels. Hence, cities (i.e., Ningbo) have defined boundaries for their in and out mobility. It is the standard model of connecting cities in terms of daily travel, commuting, and labour/worker mobility. Hence, once they are closed, the intra-mobility between cities could be limited or temporarily non-operational. This scenario occurred in the first few weeks of the pandemic across many cities and provinces in China. The measures were in place to reduce the disease spread and contain the pandemic at the regional and country levels. However, the recurring incidences have caused further disruptions beyond the early stage of the pandemic, which are beyond the scope of this study as we only focus on the early stages to argue about the effectiveness of early lockdown measures.

The original Ningbo lockdown was implemented as follows: (1) temporary closure of road transportation networks in and out of the city, apart from those for some of the essential services and supplies, (2) Ningbo residents were allowed to go outside their buildings or compounds twice a week per household, (3) temporary closure of all public activities, (4) closure of all access to villages and smaller communities in the municipalities, (5) closure of secondary industries and secondary businesses, like restaurants, and construction sites, (6) closure of all public buildings and facilities, like museums, bars/clubs/pubs, cinemas, and other entertainment facilities, and libraries, (7) limited operations of air travel, (8) limited operations of shopping malls to only supermarkets, and (9) temporary closure of education and governmental departments.

### Datasets

There are two open-source, clean and consistence datasets including (AutoNavi Map, 2021) and DXY.DX Doctor (Covid-19 confirmed cases, 2021) that have been chosen in this research to study the impact of lockdown measures on COVID-19 pandemic prevention in Ningbo from $21^{th}$ January to $12^{th}$ October 2020.

AutoNavi is a Chinese leading digital navigation data warehouse under the Alibaba group that is widely used to study daily road transportation in China. It provides the official recorded daily road mobility data for all people using inbound and outbound roads transportation networks, including public transportation and private car use. Road transportation was the critical factor in spreading the virus in Zhejiang province during the virus outbreak, as almost all flight and train travel have been stopped. The Zhejiang citizens were only allowed to travel through roads between the cities in the province, but not to other provinces in China (Chong et al., 2019).

DXY.DX Doctor is a well-known dataset that possesses an Internet hospital vocational qualification license and provides the official number of COVID-19 confirmed cases in each city/province in China (i.e., Zhejiang province) from January 21, 2020, to October 12, 2020. Chinese Center for Disease Control and Prevention (CCDC) and the National Health Commission supports this dataset to monitor the official number of confirmed cases in the People’s Republic of China each day. In China, the COVID-19 cases are officially recorded by the governmental authorities. It is the only available and the most reliable data source that could be used for such studies. There are three levels for this data: compound, district, and urban.

This research crawls AutoNavi Map and DXY.DX Doctor datasets to collect Zhejiang’s (i.e., Ningbo) travel records and the number of COVID-19 cases. Ningbo experienced one lockdown measure in early February 2020 through which the pandemic was successfully controlled in this city. This data is used to train two machine learning models to predict the number of confirmed COVID-19 cases in Ningbo city if the lockdown measure is avoided or postponed for one, three or six months.

### Data pre-processing

This article uses Min-Max-Scaler technique (Raju et al., 2020) to normalize the road transportation and COVID-19 confirmed cases data. Min-Max maps the minimum and maximum values of a dataset into 0 and 1 respectively. It scales the other values according to the range of 0 and 1.

### Correlation method

Correlation techniques are used to measure the relationships of two or several variables aiming to figure out data dependencies (Zhi et al., 2017). This research utilises a Pearson correlation matrix (Obilor & Amadi, 2018) approach to measure the correlation between Ningbo and other cities in Zhejiang province in terms of road mobility. Moreover, the correlation of two dependent variables, i.e., road mobility and the number of COVID-19 confirmed cases, is measured and studied to address the predictor (i.e., road mobility) and target (i.e., COVID-19 confirmed cases) features. Pearson is a technique to measure the strength of the linear association between two variables. It returns a value between − 1 and + 1 to indicate the correlations. For this, zero shows no association between the variables, while a value greater than zero indicates a positive relationship and a value less than zero shows a negative association between the variables. The positive correlation means that increasing the value of one variable increases the value of another one, while the negative correlation results in the reduction of one variable if the other one increases.

### Cross validation

Cross-validation is a technique used to analyse and evaluate the statistical results to be extended for independent datasets and estimate the accuracy of the prediction model (Zhang & Yang, 2015). For this, two metrics are usually measured and evaluated: Mean Squared Error (MSE) and R-square (Zhang & Yang, 2015). Mean Squared Error calculates the average of error squares between the estimated (i.e., predicted) value and the actual value (Chai & Draxler, 2014). R-square is the ratio of SSR (Sum of squares of the regression) over SST (sum of squares total). SSR is calculated as the sum of squares of the difference between the predicted and the original data. In contrast, SST is calculated by the sum of the squares of the difference of the original data and the mean value (Chicco et al., 2021).

### Machine learning analysis

Machine learning techniques are used to discover and identify data patterns (Kourou et al., 2015). They can offer several benefits, such as data prediction and classification. This paper uses machine learning analysis to predict the behaviour of the COVID-19 outbreak according to different lockdown scheduling plans such as no action, two weeks delay (of postponed lockdown), one month delay, three months delay, and six months delay. For this, two machine learning models, including Random Forest and K-Nearest Neighbours, are trained and evaluated. The machine learning models analyse the historical time-series data to forecast the number of confirmed COVID-19 cases in the future. For this, a sliding-window method (Ram Gurung & Bostrom, 2018) is used to transform the time-series dataset into supervised learning by segmenting the time-series records as daily windows. Each machine learning model is trained by the normalised results of the actual/historical number of COVID-19 confirmed cases and mobility ratio to forecast the number of future COVID-19 confirmed cases. This research uses KNN regression and Random Forest as they are suitable for nonlinear relationships between the inputs and outputs. KNN compares the inputs with historical data and returns the corresponding outputs in history. It is easy to implement and works well in some very complicated processes. Random Forest is more sophisticated in nonlinear regression (Cosenza et al., 2021) and can perfectly fit the input-output relationship with unlimited high complexity. The dataset is randomly divided into the train (80%) and test (20%) partitions to train and test the models.

**Fig. 2:**
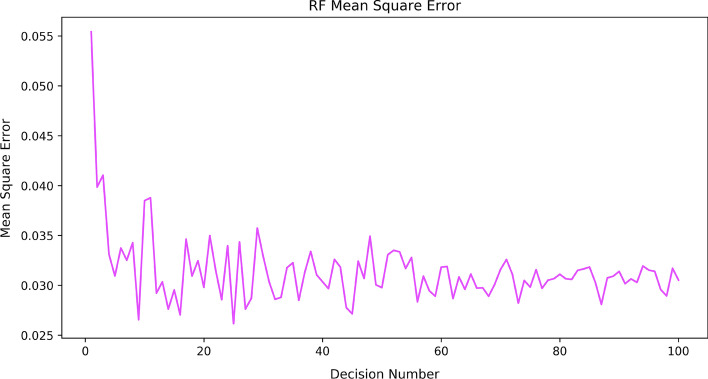
RF mean squared error

Random Forest regression operates by constructing a large number of decision trees during training to return an average prediction value (Friedman et al., 2001). This collection of decision trees measures the average/majority vote of trees as the predicted output. The advantage of Random Forest regression is to reduce the risk of over-fitting by averaging the results of multiple decision trees (Segal, 2004). Random Forest is stable to support new data samples appearing in a single decision tree (Liaw et al., 2002). Indeed, this supports ensemble methods that provide better accuracy than single prediction algorithms, especially in large datasets (Alehegn et al., 2019). The accuracy of the Random Forest regression is enhanced if the number of decision trees gradually increases. For example, Cosenza et al. (2021) reports that error rates become stable in Random Forest regression when the number of trees is close to 500. This research trains and evaluates a Random Forest model using the training dataset to find the best-fitted number of decision trees. The mean squared error of the Random Forest model is measured according to the given dataset when the number of decision trees is increased from 1 to 100. According to Fig. 2, the Random Forest model with 25 trees is the best one as it gives the minimum mean squared error.

**Fig. 3:**
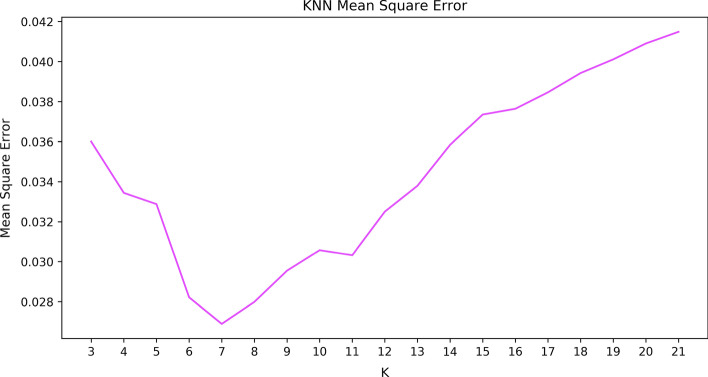
KNN mean squared error

K-Nearest Neighbours (KNN) is a non-parametric regression and example-based delayed learning algorithm. It does not assume the distribution of the data for model training. It can quickly learn complex objective functions without losing information (Goyal et al., 2014). KNN calculates the value of the corresponding attributes (i.e., prediction target) in a data sample by finding the best-fitted neighbouring parameters (i.e., K value) and assigning the average value of specific attributes of these neighbours to it (Biau et al., 2012). The accuracy of the KNN model reaches a peak (i.e., elbow) and then gradually drops if neighbouring parameters are increased (Song et al., 2017). Our KNN model is trained and evaluated using the training dataset to find the best-fitted number of neighbours (K value). This model is tested with a range of K values (3 to 20) to find the K value that gives the minimum squared error. As Fig. 3 shows, the K-Nearest Neighbours regression model with the K value of 7 has the minimum mean squared error.

## Results and discussion

This section reports and discusses the results of correlation analysis, prediction, and cross validation.

### Correlation analysis

This research aims to analyse the correlation between Ningbo’s inbound mobility and the number of COVID-19 confirmed cases using the Pearson technique (Zhi et al., 2017). It focuses on the mobility feature as this is recognised as the number one cause of disease outbreak spread (Cheshmehzangi, 2020). This fact has been evident during the COVID-19 pandemic since its inception, indicating how larger-scale mobility could speed up the process of disease spread. Later on in the pandemic, the reoccurring smaller outbreaks also highlight the impact of mobility and the development of infected clusters in cities. The correlation between mobility and the number of cases is directly linked to cases like Ningbo, where the pandemic led to one lockdown period to date. The lockdown only occurred when the number of cases increased daily and there were no restrictions on inbound and outbound mobility. The case is unique but representative of the aim of the study, highlighting the importance of mobility control during the outbreak events, such as the ongoing COVID-10 pandemic. There are, of course, other factors that could lead to disease outbreak spread, but the impact of human mobility networks (Bajardi et al., 2021; Merler & Ajelli, 2009; Cartenì, 2020)is identified to be the most predominant factor. Existing studies of mobility-based modelling of the outbreak spread imply a correlation between higher infection rate and mobility rates (Hao et al., 2020; Siwiak, 2020; Fakir & Bharati, 2021, Kartal et al., 2021). For cases like Ningbo, where only one lockdown period is implemented, the city has continued to control in and out mobility, checking individuals’ travel records and health codes.

**Table 1:** Average population and Ningbo’s inbound mobility for Zhejiang cities from January to October 2020

**City**	** Population**	**Average Ningbo’s inbound mobility (normalized)**	**Ningbo’s inbound mobility (percentage)**
Hangzhou	10,360,000	0.35	13%
Ningbo	9,404,283	N/A	N/A
Jiaxing	4,501,657	0.21	8%
Wenzhou	9,190,000	0.34	13%
Taizhou	5,968,838	0.29	11%
Shaoxing	4,912,239	0.36	13%
Jinhua	5,361,572	0.35	13%
Huzhou	2,893,542	0.20	7%
Lishui	2,506,600	0.22	9%
Quzhou	2,578,100	0.10	4%
Zhoushan	1,152,000	0.22	8%

Table 1 shows the average population and Ningbo’s Inbound Mobility normalised value and percentage from $21^{th}$ January to $12^{th}$ October 2020 in Zhejiang (COVID-19 2021). The data shows that Shaoxing, Jinhua, Taizhou, Wenzhou, and Hangzhou cities have a larger mobility ratio to Ningbo than other cities due to their geographical location and population density. For example, 13% of Shaoxing’s population travelled to Ningbo by road, whereas only 4% of Quzhou’s residents visited Ningbo from January to October 2020. It is used to study the correlations between the population of Zhejiang cities and outbound road mobility to Ningbo. As this table shows, there is no linear correlation between the city population and road mobility. For example, Shaoxing has the highest mobility to Ningbo, whereas this is the sixth city in Zhejiang province (i.e., in terms of population). This fact supports that geographical location and road distance are important factors to impact Ningbo’s inbound road mobility (Grove, 2016).

**Fig. 4:**
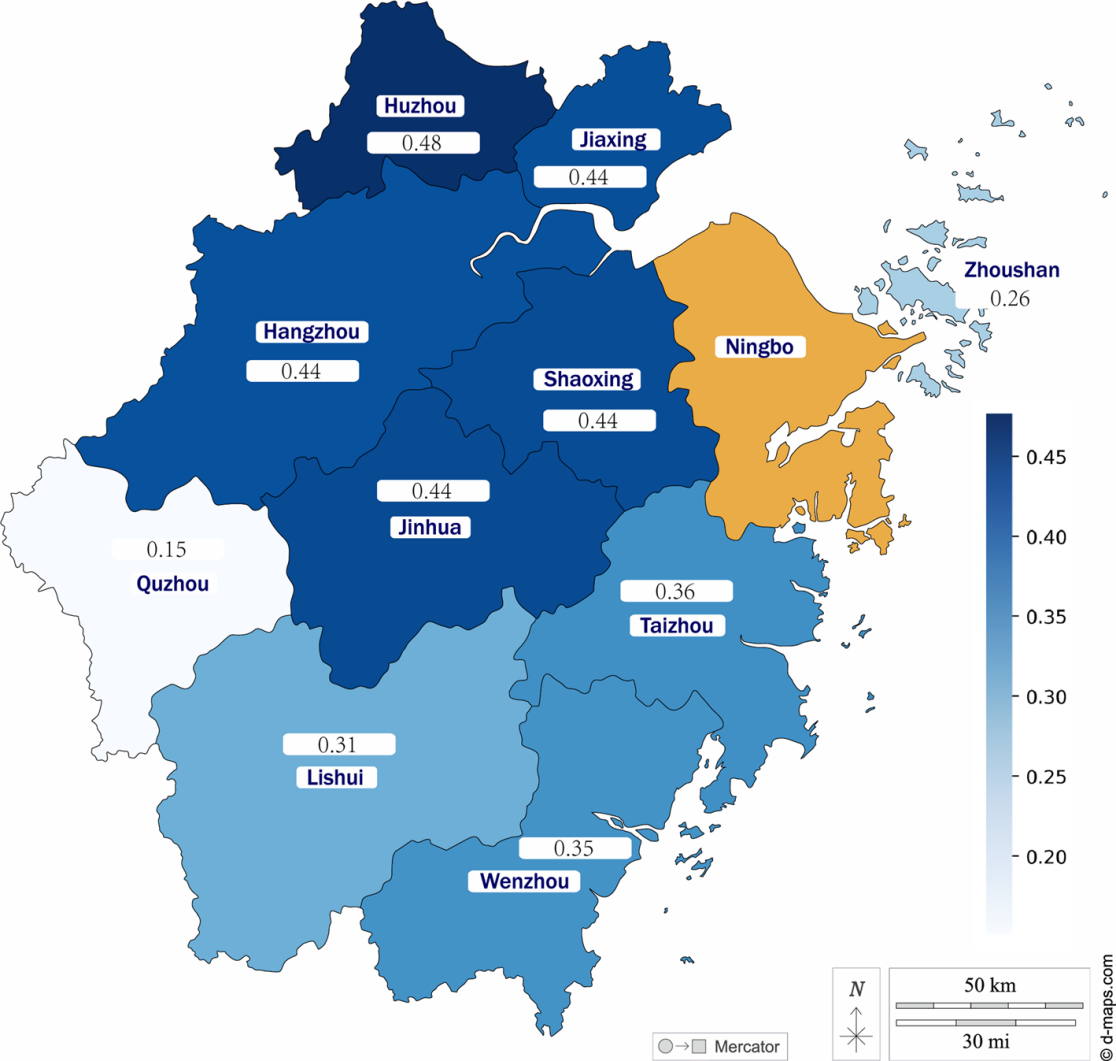
The correlation between Zhejiang cities’ mobility ratio and Ningbo’s COVID-19 cases [For example, the correlation score between Hangzhou’s mobility to Ningbo and Ningbo’s COVID-19 cases is 0.44]

Figure 4 highlights the correlation between Zhejiang cities’ mobility and Ningbo’s COVID-19 cases, which is calculated using the Pearson technique. According to this, Huzhou has the highest correlation (0.48) with the COVID-19 outbreak in Ningbo, whereas Quzhou addresses the minimum correlation score (0.15). The data supports that passengers increase the virus spread in Ningbo from closer cities with more COVID-19 cases.

### Prediction analysis

This article aims to predict the impact of the lockdown schedule on the COVID-19 outbreak using two machine learning techniques, including Random Forest and K-Nearest Neighbors (KNN). According to the results, both the machine learning models, including KNN and Random Forest, predict that the confirmed cases are sharply decreased according to each lockdown plan.

**Fig. 5:**
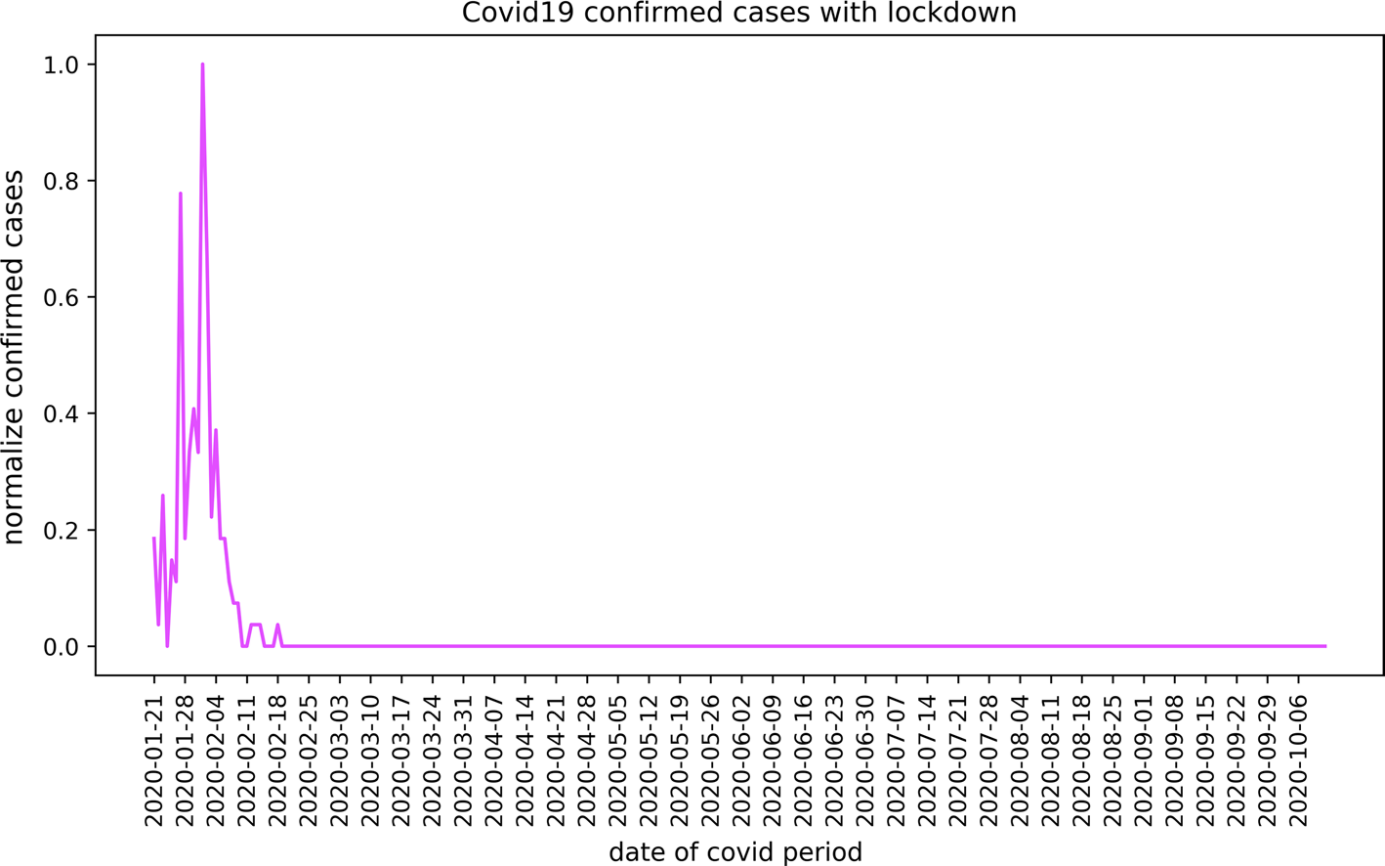
The number of COVID-19 confirmed cases in Ningbo

Figure 5 shows the normalised number of COVID-19 confirmed cases in Ningbo from January 21, 2020, to October 12, 2020. According to this data, the Zhejiang government started to take lockdown measures to restrict road transportation in early February 2020. It was an immediate lockdown measure after the pandemic outbreak and scheduled for about 45 days, until early March 2020 (Chong et al., 2019). As the Figure shows, this lockdown successfully controlled the pandemic in Ningbo as the number of confirmed cases sharply dropped after three weeks.

**Fig. 6:**
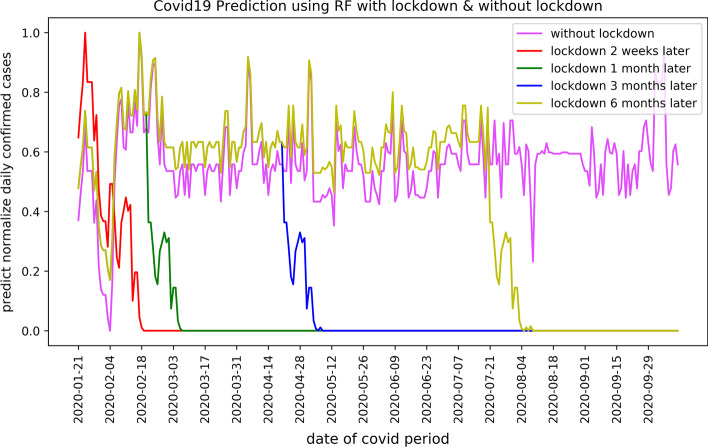
Predicted COVID-19 confirmed cases using Random Forest

*Random Forest*: Figure 6 depicts Random Forest prediction results of COVID-19 confirmed cases from January 21, 2020, to October 12, 2020, in Ningbo according to the proposed five lockdown scenarios. The findings support the efficiency of lockdown measures to control and prevent contagion, reducing the number of confirmed cases. Moreover, the results show that taking early lockdown actions (i.e., 2 weeks or one month) significantly reduces the number of COVID-19 cases than late lockdown (i.e., 6 months). According to the results, the number of COVID-19 cases reaches one or several picks before the lockdown and sharply drops after 2-week since a lockdown starts. Random Forest prediction supports a decreased number of COVID-19 confirmed cases during the national holidays (i.e., new year) in China as road mobility is reduced. However, the number of COVID-19 cases increases if the lockdown measure is avoided.

**Fig. 7:**
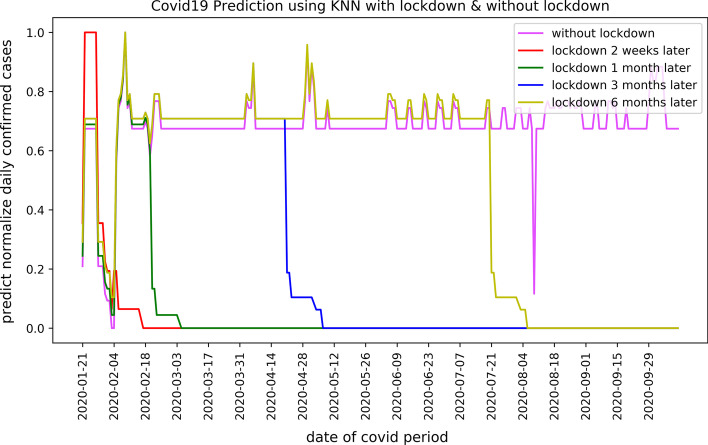
Predicted COVID-19 confirmed cases using KNN

*K-Nearest Neighbours (KNN)*: Figure 7 shows and evaluates the prediction results of K-Nearest Neighbours for the number of COVID-19 confirmed cases from January 21, 2020, to October 12, 2020, in Ningbo. Similar to Random Forest, KNN predicts the behaviour of COVID-19 outbreak according to five lockdown plans. According to the results, the number of confirmed cases sharply decreases for each lockdown plan and remains under control. This finding supports that taking an appropriate and early lockdown schedule can minimise the number of COVID-19 confirmed cases. According to the results, the number of COVID-19 cases experienced a drop in early August 2020 because of a national holiday in China and reduced road transportation.

### Cross-validation results

According to Cosenza et al. (2021), this is recommended to run cross-validation ten times. For this, the average of the absolute value errors is calculated to address the quality of a machine learning model. MSE should be minimized to reduce the loss function during regression and increase the accuracy of the prediction model, whereas R-square is a value between 0 and 1 that should be increased to address a fitted machine learning model according to the given dataset. By this, R-square ideally should be 1 if the model is the best-fitted model, and this is 0 if the model does not match the dataset (Onyutha 2020). For this, the dataset is randomly divided into the train (%80) and test (%20) partitions and MSE and R-square are measured for both Random Forest and KNN machine learning models as Table 2 shows.

**Table 2:** Accuracy for three models

**Method**	Mean squared error	R-square
K-Nearest Neighbours	0.0891	0.1417
Random Forest	0.0556	0.4646

According to 10-fold cross-validation results, the Random Forest model outperforms KNN in terms of prediction accuracy. The results show that KNN addresses a greater MSE than Random forest, while the Random Forest model shows a greater R-square than KNN. This finding supports that the Random Forest model better fits the dataset to predict the target (number of COVID-19 cases). It shows that Random Forest is sensitive to correct tuning and addresses better results than KNN, especially when the dataset is restricted and noisy.

## Conclusion and future work

COVID-19 disease is still spreading fast worldwide, and every day, many new confirmed cases and/or death cases are reported. Therefore, governments and scientists are highly interested in finding the best-fitted approach to control and prevent the pandemic. City lockdown is one of the key measures to control pandemics, such as the COVID-19 outbreak. However, lockdown is not the best policy, especially in populous countries and/or crowded cities, as it needs additional resources to manage the mobility restrictions. Yet, smart and blended working approaches can be used as a potential alternative where it is possible.

This research highlights the correlation between road transportation and COVID-19 confirmed cases and uses machine learning techniques, including Random Forest and KNN, to predict the impact of five different lockdown scenarios on COVID-19 behaviour. According to Ningbo case study in China, an accurate sceduling plan improves the impact of lockdown measures. The machine learning cross-validation results show that Random Forest addresses better prediction accuracy than KNN. Besides, the prediction results support that lockdown measures can effectively reduce the number of COVID-19 confirmed cases. They also highlight that immediate mobility restriction via early lockdown measures reduce the cognation risks of the COVID-19 virus.

The study has limitations, which are managed as part of the study’s scope. The first limitation is limited data availability, but the study uses the most accurate data to develop a baseline model/scenario. The second limitation is related to the focused scope of the study, which intentionally considers a limited number of variables. It is done to have better accuracy of results from the available data. Otherwise, the correlation analysis fails to represent what the study aims to discuss. The third limitation is based on the study context, which focuses on one selected case study instead of multiple cases or a larger-scale regional study. Nonetheless, as part of the study’s scope, this approach helps to have a more evidence-based discussion on the correlation between mobility restriction, time, and outbreak spread. For future research, such an approach could be studied at larger scales, covering regions and sub-regions, verifying the effectiveness of travel or mobility restrictions for outbreak spread control. This approach could also be applied at a much larger scale to avoid future pandemics.

Some possible improvements can be made for future research studies in the same or similar areas. This research can be extended by using a larger worldwide dataset to study the impact of lockdown measures on COVID-19 behaviour. Moreover, the impact of air/rail/sea and road transportation and the role of wearing masks and social distancing in the COVID-19 contagion could be studied further. In addition, it is suggested to use multi-target machine learning prediction techniques (i.e., neural networks and SVM) to predict multiple parameters and find the best-fitted prediction models for the COVID-19 spread and impact on the city scale.

## Data Availability

All data/materials used and/or analysed during the current study are available from the corresponding author on request.

## Footnotes

1 China territorial divisions are defined based on provincial and municipal boundaries managed by the provincial, municipal, and local governments. In China, municipalities have set territorial boundaries. A major industrial city like Ningbo includes both urban and non-urban areas under its municipality. The city is also a prefecture-level and sub-provincial city, which reflects the fact that it has six urban districts, two satellite county-level cities, and two rural countries.
